# Accuracy of Self-Reported COVID-19 Vaccination Status Compared With a Public Health Vaccination Registry in Québec: Observational Diagnostic Study

**DOI:** 10.2196/44465

**Published:** 2023-06-16

**Authors:** Patrick M Archambault, Rhonda J Rosychuk, Martyne Audet, Rajan Bola, Shabnam Vatanpour, Steven C Brooks, Raoul Daoust, Gregory Clark, Lars Grant, Samuel Vaillancourt, Michelle Welsford, Laurie J Morrison, Corinne M Hohl, Canadian COVID-19 Emergency Department Rapid Response Network (CCEDRRN) investigators, Network of Canadian Emergency Researchers, Canadian Critical Care Trials Group

**Affiliations:** 1 Department of Family Medicine and Emergency Medicine Université Laval Québec, QC Canada; 2 Centre de recherche intégrée pour un système de santé apprenant en santé et services sociaux Centre intégré de santé et services sociaux de Chaudière-Appalaches Lévis, QC Canada; 3 VITAM - Centre de recherche en santé durable Québec, QC Canada; 4 Department of Anesthesiology and Intensive Care Université Laval Québec, QC Canada; 5 Department of Pediatrics, Faculty of Medicine & Dentistry University of Alberta Edmonton, AB Canada; 6 Department of Emergency Medicine Faculty of Medicine University of British Columbia Vancouver, BC Canada; 7 Center for Health Informatics, Cumming School of Medicine University of Calgary Calgary, AB Canada; 8 Department of Emergency Medicine Queen's University Kingston, ON Canada; 9 Department of Family Medicine and Emergency Medicine Université de Montréal Montréal, QC Canada; 10 Department of Emergency Medicine, Faculty of Medicine McGill University Montréal, QC Canada; 11 Lady Davis Institute for Medical Research Jewish General Hospital Montréal, QC Canada; 12 Department of Emergency Medicine, Li Ka Shing Knowledge Institute St Michael's Hospital, Unity Health Toronto Toronto, ON Canada; 13 Department of Medicine University of Toronto Toronto, ON Canada; 14 Division of Emergency Medicine, Department of Medicine McMaster University Hamilton, ON Canada; 15 Division of Emergency Medicine, Department of Medicine University of Toronto Toronto, ON Canada; 16 Department of Emergency Services, Sunnybrook Health Sciences Centre Toronto, ON Canada; 17 University of British Columbia Vancouver, BC Canada; 18 Canadian Association of Emergency Physicians Ottawa, ON Canada; 19 Canadian Critical Care Trials Group Toronto, ON Canada

**Keywords:** electronic vaccination registry, self-reported vaccination status, COVID-19, accuracy, diagnostic study, interrater agreement

## Abstract

**Background:**

The accuracy of self-reported vaccination status is important to guide real-world vaccine effectiveness studies and policy making in jurisdictions where access to electronic vaccine registries is restricted.

**Objective:**

This study aimed to determine the accuracy of self-reported vaccination status and reliability of the self-reported number of doses, brand, and time of vaccine administration.

**Methods:**

This diagnostic accuracy study was completed by the Canadian COVID-19 Emergency Department Rapid Response Network. We enrolled consecutive patients presenting to 4 emergency departments (EDs) in Québec between March 24, 2020, and December 25, 2021. We included adult patients who were able to consent, could speak English or French, and had a proven COVID-19 infection. We compared the self-reported vaccination status of the patients with their vaccination status in the electronic Québec Vaccination Registry. Our primary outcome was the accuracy of the self-reported vaccination status (index test) ascertained during telephone follow-up compared with the Québec Vaccination Registry (reference standard). The accuracy was calculated by dividing all correctly self-reported vaccinated and unvaccinated participants by the sum of all correctly and incorrectly self-reported vaccinated and unvaccinated participants. We also reported interrater agreement with the reference standard as measured by unweighted Cohen κ for self-reported vaccination status at telephone follow-up and at the time of their index ED visit, number of vaccine doses, and brand.

**Results:**

During the study period, we included 1361 participants. At the time of the follow-up interview, 932 participants reported at least 1 dose of a COVID-19 vaccine. The accuracy of the self-reported vaccination status was 96% (95% CI 95%-97%). Cohen κ for self-reported vaccination status at phone follow-up was 0.91 (95% CI 0.89-0.93) and 0.85 (95% CI 0.77-0.92) at the time of their index ED visit. Cohen κ was 0.89 (95% CI 0.87-0.91) for the number of doses, 0.80 (95% CI 0.75-0.84) for the brand of the first dose, 0.76 (95% CI 0.70-0.83) for the brand of the second dose, and 0.59 (95% CI 0.34-0.83) for the brand of the third dose.

**Conclusions:**

We reported a high accuracy of self-reported vaccination status for adult patients without cognitive disorders who can express themselves in English or French. Researchers can use self-reported COVID-19 vaccination data on the number of doses received, vaccine brand name, and timing of vaccination to guide future research with patients who are capable of self-reporting their vaccination data. However, access to official electronic vaccine registries is still needed to determine the vaccination status in certain susceptible populations where self-reported vaccination data remain missing or impossible to obtain.

**Trial Registration:**

Clinicaltrials.gov NCT04702945; https://clinicaltrials.gov/ct2/show/NCT04702945

## Introduction

### Background

Since the beginning of the COVID-19 pandemic, vaccination campaigns have been instrumental in preventing mortality and morbidity related to COVID-19 worldwide [1]. With over 13 billion doses of vaccines administered worldwide [2], public health policy makers around the world have been relying on evidence from clinical trials to guide decision-making and develop vaccination strategies to prevent the spread and consequences of this pandemic [3]. In addition to evidence from controlled clinical trials, real-world evidence regarding vaccine effectiveness is important to guide policy making [4,5] and patient decision-making [6]. In particular, real-world evidence can help understand vaccine effectiveness against different disease severities in different susceptible populations often excluded from clinical trials [7], inform policy makers about the length of vaccine protection over time [8], and document rarer side effects [9]. Real-world data can also help understand the evolving prevalence and underlying causes of vaccine hesitancy over time [10] and shape future vaccination campaigns [11,12]. Understanding the role of herd immunity and how to promote vaccination are important questions that need reliable vaccination data [13-16]. Studying COVID-19 occupational hazards for professionals working with susceptible populations also requires timely and reliable access to vaccination data [17,18]. As new variants emerge, real-world evidence informs current and future strategies to protect against COVID-19 infection [19]. To measure real-world vaccine effectiveness and inform evidence-based health promotion strategies, researchers and policy makers alike need access to reliable vaccination data.

Unfortunately, not all researchers and policy makers have access to official electronic public health vaccine registries [20-23], while others have substantial challenges in securing access to individual-level vaccination data even when they exist [24]. Many researchers and policy makers must then rely on patients’ self-reported status to measure real-world vaccine effectiveness, and if this is inaccurate, it may bias vaccine effectiveness estimates in test-negative designs [25]. For these studies, not only are accurate data about vaccination status key, but, most importantly, reliable data about when vaccine doses were administered are essential to determine the effectiveness in preventing infections and health service use such as emergency department (ED) visits [26].

### Objective

To inform the conduct of real-world vaccine effectiveness studies using patient self-reported vaccine information and to support the development of evidence-based public health promotion policies, this study aimed to compare the accuracy of self-reported vaccination status for COVID-19 with data from an official electronic vaccination registry in the province of Québec, Canada [27].

## Methods

### Study Design and Setting

The Canadian COVID-19 Emergency Department Rapid Response Network (CCEDRRN; pronounced “SED-rin”) is a national collaboration created to harmonize data collection related to COVID-19 in over 50 EDs across 8 provinces in Canada (British Columbia, Alberta, Manitoba, Saskatchewan, Ontario, Québec, New-Brunswick, and Nova Scotia) [28]. The CCEDRRN has created a national observational database of suspected and confirmed patients with COVID-19 who presented to acute care EDs. Data from this network are used in national studies to inform decision-making, emerging treatment guidelines, resource allocation, and planning in response to the pandemic. As of April 2023, CCEDRRN had captured data on over 208,000 patients across Canada [29]. In December 2020, CCEDRRN initiated data collection on vaccination status that supports this study.

This CCEDRRN study is a nested diagnostic accuracy study within the larger CCEDRRN cohort that was reported using the 2015 Standards for Reporting of Diagnostic Accuracy studies [30]. This study only used CCEDRRN data collected from 4 sites in the province of Québec because access to government electronic vaccine registries in the other provinces of Canada was unavailable. These 4 Québec CCEDRRN sites only collected data on patients with a positive COVID-19 test. The study enrolled consecutive consenting patients presenting to these 4 participating EDs between March 24, 2020, and December 25, 2021.

Vaccination in Québec started on December 14, 2020, with long-term care residents and health care workers [31]. On March 1, 2021, vaccination in the general population started in sequence based on their age group. On April 14, 2021, more than 2 million Québecers had received at least 1 dose, representing a quarter of the population. On May 18, 2021, more than half of the population had received at least 1 dose, and on June 6, more than 75% of the population aged >12 years had received at least 1 dose. On July 5, 2021, Québec reduced the dosing interval to 4 weeks to maximize 2-dose coverage, such that by September 30, 2021, a total of 75% of adults were considered fully vaccinated. On September 28, 2021, a third booster dose was recommended for residents in long-term care and assisted living. Between November 16, 2021, and December 29, 2021, the booster dose was progressively authorized for specific high-risk subgroups and then in sequence for the general population based on age groups. After each dose was administered in Québec, vaccinators were required to document the vaccine brand and the time of administration directly within the provincial electronic registry [32]. The identities of the vaccinated patients were validated using at least 2 personal identification questions. Another key public health measure to consider for this study was the mandate on September 1, 2021, to implement a mandatory vaccination passport that contained information about the number of doses, vaccine brand names, and vaccination dates to access certain public services in Québec [31].

### Ethics Approval and Informed Consent

The ethics committee at the *Centre intégré de santé et de services sociaux de Chaudière-Appalaches* approved this project in February 2021 (MP-23-2021-766) with a waiver of informed consent for retrospective data collected; however, informed consent was obtained during telephone follow-up for all participants. The participants did not receive any financial compensation.

### Participants

Patients were included if they had a confirmed COVID-19 infection (≥1 nucleic acid amplification tests positive for SARS-CoV-2 from specimens collected during the index ED visit, 14 days before the ED visit in the community, or within 14 days after admission) [28]. Included patients were aged ≥18 years, were able to communicate in French or English, and consented independently to telephone follow-up and the use of their health data. All included patients sought care at any of the 4 participating Québec sites (Hôtel-Dieu de Lévis, Hôpital Sacré-Coeur de Montréal, Royal Victoria Hospital, and the Montreal General Hospital). Patients were excluded if they were unable to provide consent (eg, had died since the ED encounter), were unable to communicate in French or English, could not be reached after 5 attempts, or did not have a record in the Québec Vaccination Registry (eg, out-of-province patients).

### Data Collection and Outcome Measures

Trained research assistants collected data for our study from different sources and at different time periods. First, starting on July 9, 2020, we used a retrospective chart review to extract demographics, the time and date of ED visits, chief complaint, laboratory tests, treatments, admission status, and comorbidities. We retrospectively collected data for the index visits of all patients and any subsequent ED visits at least 30 days after the index visit. Second, starting on March 24, 2021, we initiated data collection from the Québec Vaccination Registry and phone follow-ups. A single research assistant at each site was designated to access the Québec Vaccination Registry to determine patients’ vaccination status, vaccine brands received, the total number of doses, and dates of vaccination. Separate research assistants at each site were trained to only conduct telephone follow-ups. They were blinded to the reason for collecting self-reported vaccination data and did not access the information collected from the Québec Vaccination Registry. These research assistants contacted patients by telephone to obtain consent to use their data; to obtain additional sociodemographic data not available in their medical records (eg, income, self-identified race, and education level); and to collect their self-reported vaccination data (vaccination status, vaccine brands received, the total number of doses, and dates of vaccination). The median (IQR) time to contact the patients was 202 (108-352) days after the ED index visit. As patients were called at most 30 days after their data were consulted from the Québec Vaccination Registry, we only used self-reported vaccination data from before the date we consulted the Québec Vaccination Registry.

In accordance with CCEDRRN’s national data management plan, after assigning a unique study identifier for each participant, deidentified data were collected and stored in REDCap (Research Electronic Data Capture; Vanderbilt University) at the University of British Columbia. After data verification, registry data were uploaded into CaraSpace, a secure private cloud for the storage and analysis of privacy-sensitive data [28]. Analysts accessed the cloud space via an encrypted virtual private network through a firewall and 2-factor authentication.

The primary outcome of this study was the accuracy of self-reported COVID-19 vaccination status at the time of the telephone follow-up compared with their vaccination status in the Québec Vaccination Registry. We considered the self-reporting of vaccination status via telephone as the index test and the Québec Vaccination Registry as the reference standard. The index test had 2 possible results: “no vaccine dose” (self-reported as not vaccinated) or “at least one vaccine dose” (self-reported as vaccinated). We calculated the accuracy of the self-reported vaccination status as follows:

Accuracy = (correctly self-reported as vaccinated + correctly self-reported as not vaccinated) / (correctly self-reported as vaccinated + correctly self-reported as not vaccinated + incorrectly self-reported as vaccinated + incorrectly self-reported as not vaccinated) **(1)**

We also compared the clinical and demographic characteristics of patients with concordant and discordant self-reported vaccination status compared with the Québec Vaccination Registry.

Secondary outcomes were the sensitivity, calculated as


(correctly self-reported as vaccinated / [correctly self-reported as vaccinated + incorrectly self-reported as not vaccinated]) **(2)**

and specificity, calculated as


(correctly self-reported as not vaccinated / [correctly self-reported as not vaccinated + incorrectly self-reported as vaccinated]) **(3)**

of the self-reported vaccination status and vaccination status in the Québec Vaccination Registry. Relaxing the reference standard assumption about the Québec Vaccination Registry, we also measured the interrater agreement between self-reported vaccination status and vaccination status in the Québec Vaccination Registry, interrater agreement between self-reported COVID-19 vaccine brand received and the brand recorded in the Québec Vaccination Registry, and interrater agreement between self-reported number of COVID-19 vaccine doses received compared with the number of doses recorded in the vaccine registry.

An additional secondary outcome was the interrater agreement between vaccination status at the time of the index ED visit determined retrospectively by using the self-reported vaccination date compared with the vaccination status at the time of the index ED visit determined by the vaccination date in the Québec Vaccination Registry (Multimedia Appendix 1). Vaccination status was defined as fully, partially, and not vaccinated at the time of the index ED visit based on the number of doses and time since the last dose received to account for likely waning protection [33,34] (Multimedia Appendix 1). We defined fully vaccinated patients at the time of the index ED visit as patients who received ≥2 doses ≥14 days before the index ED visit but less than 6 months since the last dose. We defined partially vaccinated patients as having received only 1 dose before the index ED visit or having received their second or third dose <14 days before the index ED visit or having received their second or third dose >6 months before the index ED visit. Patients were considered not vaccinated if no doses had been received before their index ED visit. Patients who could not recall the date of their last vaccination were excluded from this analysis.

### Analysis

The vaccination data in the Québec Vaccination Registry were considered the reference standard. As the interest was to compare the same information collected by telephone as in the Québec Vaccination Registry, the sample size calculations were based on a 1-sided binomial test for sensitivity [35,36]. A sample size of 613 patients was needed to achieve a 95% CI for sensitivity of half width 0.02 assuming a sensitivity of 0.98 and prevalence of at least 0.40.

Data were summarized by using descriptive statistics (eg, mean [SD], counts, and percentage). Accuracy, sensitivity, and specificity for COVID-19 vaccination status reported by patients compared with the reference standard (ie, the Québec Vaccination Registry) were calculated with associated 95% CIs. Secondary analyses relaxed the reference standard assumption and provided interrater agreement using the unweighted Cohen κ coefficient [37]. We chose to use the unweighted Cohen κ because we had 2 raters (ie, self-report by participant and report from the Québec Vaccination Registry), and we wanted disagreements to carry the same weight. All analyses were conducted in R (R Foundation for Statistical Computing) using the *rms* and *psych* packages [38-40]. To ensure patient privacy, a cell-size restriction policy prohibited us from reporting counts of <5.

## Results

### Participant Characteristics

**Figure 1:**
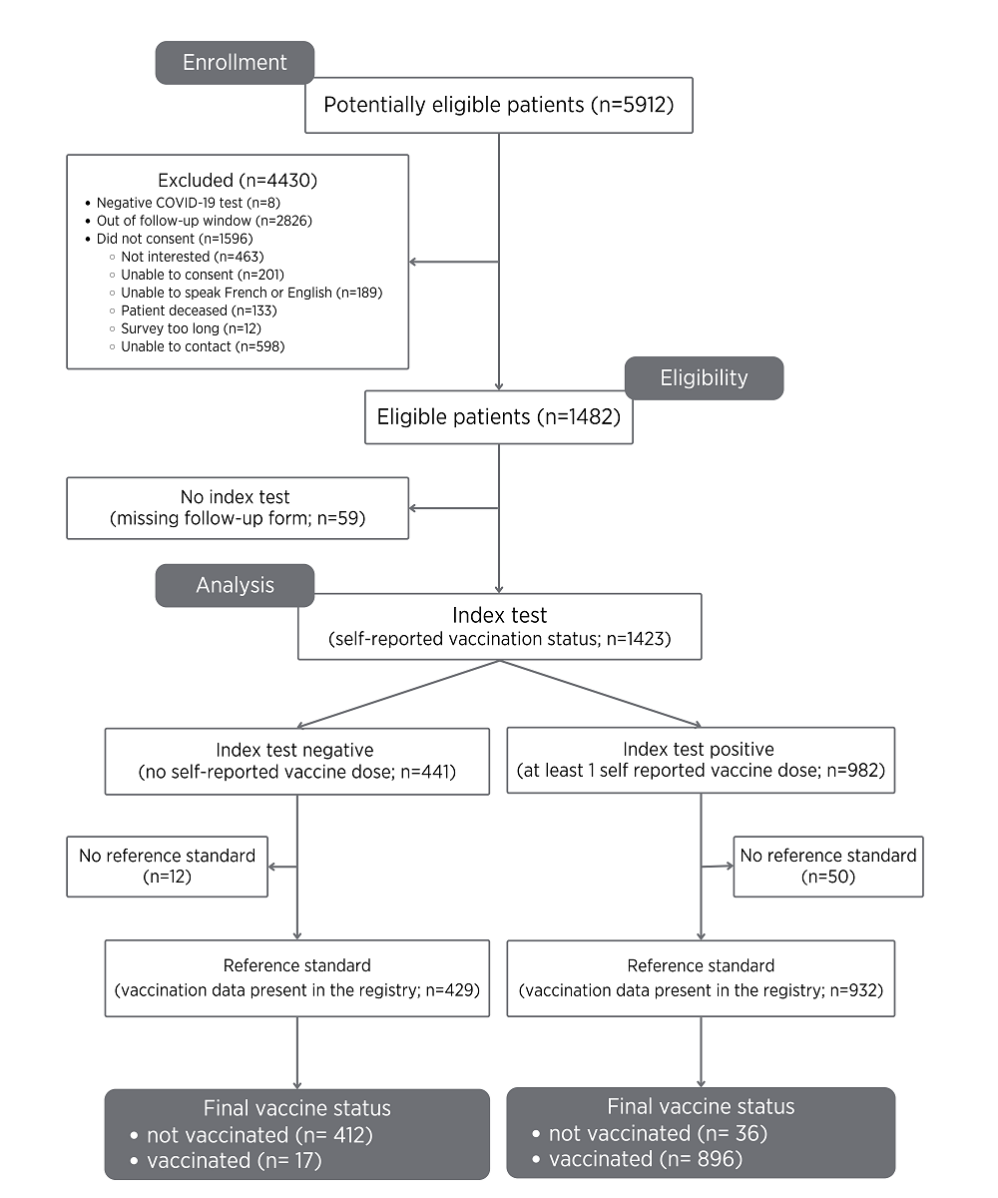
Flow diagram of patients in the study.

During the study period, there were 5912 adult patients who presented to the Québec study sites, and 1482 met the inclusion criteria; of them, 1361 (23.02%) patients had the required data from follow-up and available in the Québec Vaccination Registry (Figure 1). Among the 4430 excluded patients, 1596 (36.03%) did not consent to participate or were not able to consent. There were also 62 (1.05%) patients who did not have any vaccination record in the Québec Vaccination Registry because they were patients from outside the province or did not have a provincial health insurance number. The baseline participant characteristics are shown in Table 1. Patients who did not or could not consent were slightly older than patients who participated (age: mean 58.6, SD 21.3 years vs mean 55.1, SD 17.4 years, respectively) and were more likely to be male individuals (nonconsenting male individuals: 871/1596, 54.57%, vs consenting male individuals: 686/1361, 50.4%). Three-quarters (1010/1361, 74.21%) of the patients presented to the ED during the second and third waves of the pandemic (August 23, 2020, to July 17, 2021).

**Table 1:** Sociodemographic and clinical characteristics of study participants whose index visit occurred in a Québec emergency department between March 24, 2020, and December 25, 2021 (n=1361).

			Study participants
Age (years), mean (SD)			55.1 (17.4)
**Age group (years), n (%)**			
	<24	45 (3.3)	
	25-39	230 (16.9)	
	40-64	685 (50.3)	
	65-79	272 (20)	
	≥80	129 (9.5)	
**Sex, n (%)**			
	Male	686 (50.4)	
	Female	675 (49.6)	
	Intersex	0 (0)	
**Site, n (%)**			
	Hôtel-Dieu de Lévis	293 (21.5)	
	Royal Victoria	501 (36.8)	
	Montreal General	132 (9.7)	
	Sacré-Coeur de Montréal	435 (32)	
**Index visit occurred during, n (%)**			
	Wave 1 (March 1, 2020, to August 22, 2020)	207 (15.2)	
	Wave 2 (August 23, 2020, to March 20, 2021)	725 (53.3)	
	Wave 3 (March 21, 2021, to July 17, 2021)	285 (20.9)	
	Wave 4 to early 5 (July 18, 2021, to December 31, 2021)	144 (10.6)	
**Index visit occurred before the start of vaccination campaign in Québec** **(December 14, 2020),** **n (%)**			
	Prevaccination campaign	490 (36)	
	Postvaccination campaign	871 (64)	
Time elapsed from index visit to telephone follow-up (days), median (IQR)			202 (108-352)
**Top 10 comorbid conditions, n (%)**			
	Hypertension	440 (32.3)	
	Dyslipidemia	314 (23.1)	
	Diabetes	241 (17.7)	
	Hypothyroidism	160 (11.8)	
	Asthma	144 (10.6)	
	Psychiatric condition or mental health diagnosis	113 (8.3)	
	Coronary artery disease	99 (7.3)	
	Past malignancy	73 (5.4)	
	Chronic neurological conditions	66 (4.8)	
	Rheumatologic disorder	66 (4.8)	
**ED^a^ disposition, n (%)**			
	Discharged home	740 (54.4)	
	Admitted	576 (42.3)	
	Transferred to other hospital	38 (2.8)	
	Transfer to long-term care or rehab	<5^b^ (<0.4)	
	Other	6 (0.4)	

^a^ED: emergency department.

^b^Cells with fewer than 5 individuals could not be presented as requested by the ethics committee.

### Vaccination Status

Over two-thirds (932/1361, 68.55%) of the participants reported at least 1 dose of a COVID-19 vaccine at the time of their follow-up interview (Table 2). The most administered vaccine type was Pfizer-BioNTech (689/1361, 50.6%) followed by Moderna (158/1361, 11.6%).

**Table 2:** Québec Vaccination Registry information and self-reported vaccination status during the initial telephone follow-up of participants whose emergency department index visit occurred between March 24, 2020, and December 25, 2021 (n=1361).

			Québec Vaccination Registry, n (%)		Self-reported vaccination status, n (%)
**Vaccination status**					
	Yes (at least 1 dose)	913 (67.1)		932 (68.5)	
	No (no dose given)	448 (32.9)		429 (31.5)	
	Unknown or not assessed	0 (0)		0 (0)	
**First vaccine brand**					
	Pfizer-BioNTech	707 (51.9)		689 (50.6)	
	Moderna	162 (11.9)		158 (11.6)	
	AstraZeneca or Covishield	42 (3.1)		42 (3.1)	
	Johnson & Johnson	<5 (<0.4)		<5^a^ (<0.4)	
	Unknown, but vaccinated	0 (0)		37 (2.7)	
	Unknown or not vaccinated	448 (32.9)		433 (31.8)	
**Number of vaccines doses**					
	0	448 (32.9)		429 (31.5)	
	1	366 (26.9)		368 (27)	
	2	501 (36.8)		511 (37.5)	
	3	46 (3.4)		53 (3.9)	

^a^Cells with fewer than 5 individuals could not be presented as requested by the ethics committee.

### Self-Reported Vaccination Status Accuracy and Interrater Agreement Between Self-Reported Vaccination Data and Québec Vaccination Registry Data

We reported high measures of accuracy (96%), sensitivity (98%), and specificity (92%) for self-reported vaccination status compared with the Québec Vaccination Registry as the reference standard (Tables 3 and 4). Moreover, Cohen κ was 0.91 (95% CI 0.89-0.93), indicating excellent agreement between patient self-report and the Québec Vaccination Registry.

With respect to vaccine brand type, Cohen κ for dose 1 brand was 0.80 (95% CI 0.75-0.84) indicating strong agreement (Table 5). However, this estimate decreased with subsequent doses: Cohen κ was 0.76 (95% CI 0.70-0.83) and 0.59 (95% CI 0.34-0.83) for dose 2 and dose 3 brands, respectively.

Most patients reported receiving 1 dose (368/1361, 27.04%) or 2 doses (511/1361, 37.56%) of COVID-19 vaccines at the time of the follow-up interview (Table 6). Only 3.89% (53/1361) of patients reported receiving 3 doses. One-third (429/1361, 31.52%) of the patients reported not having received the COVID-19 vaccine at the time of follow-up. Cohen κ for the number of self-reported doses compared with the Québec Vaccination Registry was very strong, at 0.89 (95% CI 0.87-0.91).

Cohen κ for self-reported vaccination status at the time of ED index visit was 0.85 (95% CI 0.77-0.92). However, many patients (774/1361, 56.87%) could not recall the exact date of their latest vaccine dose and had to be excluded from this analysis. Of those who did recall their vaccination dates, most (538/587, 91.7%) reported not being fully vaccinated at the time of the ED visit, with only 16 (2.7%) patients being fully vaccinated (Table 7).

The demographic, clinical, and sociocultural characteristics of patients with discordant (n=53) and concordant (n=1308) vaccination statuses are shown in Multimedia Appendix 2. Participants with discordant self-reported vaccination status compared to those with concordant self-reported vaccination status were younger (age: mean 51.1, SD 20.5 years, vs mean 55.3, SD 17.3 years, respectively), had lower self-reported income (5/53, 9% reported current income >CAD $73,701 (US $56,693) per year vs 294/1308, 22.48%), had lower education level (5/53, 9% with a university degree vs 444/1308, 33.94%), and had a lower vaccination rate according to the Québec Vaccination Registry (17/53, 32% vs 896/1308, 68.5%). The median time elapsed between the ED index visit and the telephone follow-up to determine the patient’s self-reported vaccination status was similar for discordant (median 94, IQR 53-191 days) and concordant cases (median 126, 62-196 days). Immigration status, race, and ethnicity did not influence the accuracy of our results.

**Table 3:** Confusion matrix comparing self-reported vaccination status with the Québec Vaccination Registry data for all participants whose emergency department index visit occurred between March 24, 2020, and December 25, 2021 (n=1361).

Self-reported vaccination status (index test)	Vaccination status in the Québec Vaccination Registry (reference standard), n	
	Yes	No
Yes	896	36
No	17	412

**Table 4:** Sensitivity, specificity and accuracy of self-reported vaccination status and interrater agreement of self-reported vaccination status compared with the Québec Vaccination Registry for all participants whose emergency department index visit occurred between March 24, 2020, and December 25, 2021 (n=1361).

	Measure (95% CI)
Sensitivity	0.98 (0.97-0.99)
Specificity	0.92 (0.89-0.94)
Accuracy	0.96 (0.95-0.97)
Cohen κ	0.91 (0.89-0.93)

**Table 5:** Interrater agreement for self-reported brand by dose compared with the Québec Vaccination Registry for all participants whose emergency department index visit occurred between March 24, 2020, and December 25, 2021.

Dose and self-reported vaccine brand		Québec Vaccination Registry vaccine brand, n					Cohen κ^a^ (95% CI)
		Pfizer	Moderna	Astra Zeneca or Covishield	Johnson & Johnson		
**1**							0.80 (0.75-0.84)
	Pfizer	645	19	<5	0		
	Moderna	11	137	0	0		
	AstraZeneca or Covishield	<5	0	38	0		
	Johnson & Johnson	0	0	0	<5		
	Unknown, but vaccinated	29	6	<5	0		
**2**							0.76 (0.70-0.83)
	Pfizer	386	19	<5	0		
	Moderna	5	84	0	0		
	AstraZeneca or Covishield	0	0	9	0		
	Unknown, but vaccinated	16	5	0	0		
**3**							0.59 (0.34-0.83)
	Pfizer	24	<5	0	0		
	Moderna	<5	7	0	0		
	Unknown, but vaccinated	<5	<5	0	0		

^a^Cohen κ calculated without participants who answered “Unknown, but vaccinated” were 0.89 (95% CI 0.86-0.93) for first dose, 0.85 (95% CI 0.80-0.91) for second dose, and 0.70 (95% CI 0.43-0.97) for third dose.

**Table 6:** Interrater agreement for self-reported number of doses compared with the Québec Vaccination Registry for participants whose emergency department index visit occurred between March 24, 2020, and December 25, 2021 (n=1361).^a^

Self-reported number of doses	Number of doses in the Québec Vaccination Registry, n			
	0	1	2	3
0	412	7	8	<5
1	20	336	12	0
2	13	23	469	6
3	<5	0	12	38

^a^Cohen κ: 0.89 (95% CI 0.87-0.91).

**Table 7:** Interrater agreement for self-reported vaccination status at emergency department index visit date versus the Québec Vaccination Registry for participants whose emergency department index visit occurred between March 24, 2020, and December 25, 2021 (n=587).^a^

Self-reported vaccination status determined using the date of latest vaccination dose in relation to the ED^b^ visit date^c^	Québec Vaccination Registry vaccination status determined in relation to the ED index visit date^d^, n		
	Not vaccinated	Partially vaccinated	Fully vaccinated
Not vaccinated	527	5	6
Partially vaccinated	<5	29	0
Fully vaccinated	0	0	16

^a^Cohen κ: 0.85 (95% CI 0.77-0.92).

^b^ED: emergency department.

^c^Categories of vaccination status at the time of ED index visit (fully, partially, and not vaccinated) for this analysis are defined in Multimedia Appendix 1.

^d^A total of 774 (56.9%) participants were excluded from this analysis because they could not recall the date of their last dose.

## Discussion

### Principal Findings

We found that the participants’ self-reported vaccination status was highly accurate at the time of the initial follow-up telephone call. The interrater agreement between the self-reported vaccine number of doses and the number of doses recorded in the vaccine registry was also strong. The agreement between self-reported vaccination status at the index visit and the patient’s vaccination status as determined by the registry was strong when patients could provide vaccination dates. However, many patients could not recall their vaccination dates. Although there was a strong and moderate agreement for the self-reported vaccine brand for the first and second doses, respectively, the agreement dropped to weak for the vaccine brand of the third dose. A few discordant cases concerning the accuracy of patients’ self-report of receiving at least 1 dose were found. The discordant cases involved patients of younger age groups, lower self-reported pre– and post–COVID-19 income, lower education levels, and lower vaccination rates in the Québec Vaccination Registry.

Our results are similar to those of a study conducted in the United States by Stephenson et al [41] that showed high levels of agreement between self-reported vaccination status and official computerized vaccination registry data (unweighted κ=0.9127, 95% CI 0.9109-0.9145). Similar to our study, they also found that discordant self-reported vaccination data were associated with lower education levels and unemployment. It could be that both studies found that lower education is associated with lower accuracy of self-reported vaccination status because information provided about vaccination status tends to be complex [42]. Comprehending and remembering this information requires certain health literacy skills [43]. Rolnick et al [44] also found that education and income level could influence the accuracy of self-reported vaccination status for 8 different vaccines (pneumococcal, influenza, tetanus diphtheria, tetanus diphtheria pertussis, human papillomavirus, hepatitis A, hepatitis B, and herpes zoster).

Contrary to our study, the study by Stephenson et al [41] found that the female sex increased the risk of discordant self-reported vaccination status. Sex and gender are important factors in understanding the real-world effectiveness of vaccines and postmarketing safety monitoring [45,46]. Sex and gender differences in vaccine hesitancy and immunization outcomes have been observed for COVID-19 and other vaccine-preventable diseases, with women typically reporting more vaccine hesitancy and higher adverse reactions than men [47-49]. Our results support the accuracy of self-reported vaccination data from both men and women.

Ulrich et al [50] studied the accuracy of the self-reported vaccination status by university students in a 2-dose meningococcal vaccine campaign. This study showed good self-reported vaccination status accuracy at 2 months (86%) and poor self-reported vaccination status at 20 months (69%). King et al [51] also reported a decrease in the agreement of patient self-reported vaccination status compared with a registry for prior season influenza vaccination (κ=0.86) versus the current season (κ=0.95). Although our study did not observe a time-dependent increase in discordant self-reported vaccination status, we did find that patients reporting a higher number of doses were more likely to forget the vaccine brand received and did not remember the dates of their latest vaccination dose. In patients who did remember the vaccine brand for each dose, the agreement with the registry was high. This limits the use of self-reported vaccination brand names for studies comparing the effectiveness of multiple doses of different types of vaccines [52].

The reliability of data on the timing of vaccination before being tested for a viral infection such as SARS-CoV-2 is essential for test-negative design vaccine effectiveness studies [53]. Our study was interested in determining the reliability of the self-reported vaccination data (ie, the date of their last vaccination and the total number of doses) used to retrospectively determine their vaccination status (not vaccinated, partially vaccinated, or fully vaccinated) at the time of their index ED visit. For patients who could remember their last vaccination date, our study found high levels of agreement between the self-reported vaccination status determined at the ED index visit date and the Québec Vaccination Registry. Unfortunately, more than half of our participants could not recall the date of their last dose, making this measure subject to an important selection bias when used in test-negative designs. This is an argument to support wider implementation and easy access to rigorous vaccination registries in all jurisdictions [21]. This is especially important to improve the care, research, and support of vulnerable populations presenting to the ED. In Canada, access to these registries is currently restricted by data custodians and administrative barriers that hinder timely knowledge creation and decision-making for a rapid-learning health system [54,55]. Although a study on the accuracy of self-reported influenza and pneumococcal vaccination status in a high-risk inflammatory bowel disease population showed high accuracy for self-reported influenza (97%) and pneumococcal (84%) vaccination status [56], our study did not find any difference between high-risk groups with different comorbidities. In concordance with Laurence et al [57], who found that older patients accurately self-reported their influenza (96%) and pneumococcal (87%) vaccine status, we also found that older adults adequately self-reported their COVID-19 vaccine status. Surprisingly, we found a higher proportion of discordant self-reported COVID-19 vaccination statuses among patients aged ≤34 years. This could be associated with the fact that COVID-19 presents a milder course in younger patients [57,58] and increased vaccination hesitancy in this age group [59-61].

There are several potential explanations for the high accuracy of self-reported vaccination status at telephone follow-up and moderate to strong agreement between the self-reported number of doses and the self-reported vaccination status determined at the time of the ED index visit. First, this high accuracy and strong agreement could have been caused by the implementation of Québec’s mandatory vaccine passport, which registered dates, brands, and doses as a requirement to access the workplace and most commercial services. Although we did not ask participants to consult their passports before answering our phone questionnaire, our study personnel did not prevent participants from consulting their passports during our telephone follow-ups. Participants’ recall of their own vaccination data could also have been improved by having to consult their passport frequently, as both federal and provincial governments in Canada granted substantial benefits to fully vaccinated citizens, such as lifting travel restrictions and access to event venues, fitness centers, and restaurants via the use of a vaccination passport [62]. Second, the impact of COVID-19 on the Canadian population has been substantial, and it may have improved the recall and the accuracy of the self-reported vaccination status. Third, the COVID-19 vaccination campaign; the omnipresence of the COVID-19 pandemic in the lay press; the many frequently reported statistics portraying the evolution of this pandemic (cases, hospitalizations, deaths, and population vaccination coverage); and the vigorous public debate about vaccination public health mandates may have also raised citizens’ awareness of their own vaccination status. Fourth, this study only included patients with confirmed COVID-19 infections. This could have improved the recall of participants compared with patients who were not infected with COVID-19.

Our results indicate that when access to official electronic vaccination registries is not available, the reliance on patients’ self-reported COVID-19 vaccination status, vaccine brand received, number of doses, and vaccination dates (when provided) can be used for research purposes. This is a substantial finding for researchers in jurisdictions that do not have access to electronic vaccine registries [20,63] and for others that have substantial challenges in securing timely access to individual-level vaccination data even when they exist [24]. Vaccine registries can also suffer from missing data for patients who have received their vaccines in different jurisdictions (as shown in our results), misclassification bias because of human error, and the timeliness of data entry into electronic vaccination registries [64]. Relying on timely and highly accurate self-reported vaccination status reduces the potential underestimation of real-world test-negative vaccine effectiveness studies introduced by vaccination-reporting bias [25]. Knowing the sensitivity and specificity of the self-reported vaccination status is also important to correct the estimation of vaccine effectiveness based on the observed risks for vaccination status misclassification [65].

This study has limitations. First, although we assumed that the Québec Vaccination Registry was the reference standard, we cannot rule out the possibility that some vaccination data were entered incorrectly. Second, our results do not apply to patients with cognitive disorders who cannot provide consent. This represents a selection bias that we could not avoid because of ethical constraints. Patients who consented to participate in our study were also more likely to be younger and female than those who did not consent or who could not consent. Understanding the impact of the pandemic on patients who cannot consent to provide their vaccination status because of cognitive issues will still necessitate access to linked vaccination registry data. Access to a vaccination registry also avoids systematic differences in the characteristics of consenting patients versus nonconsenting patients in studies that rely on self-reporting. Third, although we had many landed immigrants constituting an ethnically diverse sample, our results are potentially not applicable to patients who do not speak English or French. Other studies have shown that the self-reported vaccination status is inaccurate in certain susceptible and hard-to-follow-up racial and ethnic groups [44,50]. Although we had to exclude patients who could not speak English or French, immigration and race and ethnicity did not influence the accuracy of our results. Fourth, our study did not verify the accuracy of vaccination status provided at the time of ED care. Future prospective studies at the point of care would be needed to validate the accuracy of self-reported data for use in guiding clinical decision-making.

### Conclusions

Our study found that relying on self-reported COVID-19 vaccination status is accurate for adult patients without cognitive disorders who can express themselves in English or French. Researchers can use self-reported COVID-19 vaccination data on the number of doses received, vaccine brand name, and timing of vaccination to guide future research with certain groups of patients who are capable of self-reporting their vaccination data. However, access to official electronic vaccine registries is still needed to determine the vaccination status in certain susceptible populations where self-reported vaccination data remain missing or impossible to obtain.

## Appendix

Multimedia Appendix 1 Definitions used to determine the interrater agreement between the self-reported vaccination status and the vaccination status determined by the Québec Vaccination Registry at the time of emergency department index visit.

Multimedia Appendix 2 Characteristics of all participants by concordant or discordant vaccination status.

Multimedia Appendix 3 Contributors to the Canadian COVID-19 Emergency Department Rapid Response Network.

## Abbreviations

CCEDRRN: Canadian COVID-19 Emergency Department Rapid Response Network
ED: emergency department
REDCap: Research Electronic Data Capture

## Data Availability

Data are available on reasonable request. For investigators who wish to access Canadian COVID-19 Emergency Rapid Response Network data, proposals may be submitted to the network for review and approval by the network’s peer review publication committee, the data access and management committee, and the executive committee as per the network’s governance. Information regarding submitting proposals and accessing data may be found on the Canadian COVID-19 Emergency Department Rapid Response Network website [9].
