# Supplementary figures and images for: Accuracy of Self-Reported COVID-19 Vaccination Status Compared With a Public Health Vaccination Registry in Québec: Observational Diagnostic Study
Source: JMIR Public Health Surveill. 2023 Jun 16;9:e44465. doi: 10.2196/44465 (PMC10278735; doi:10.2196/44465)

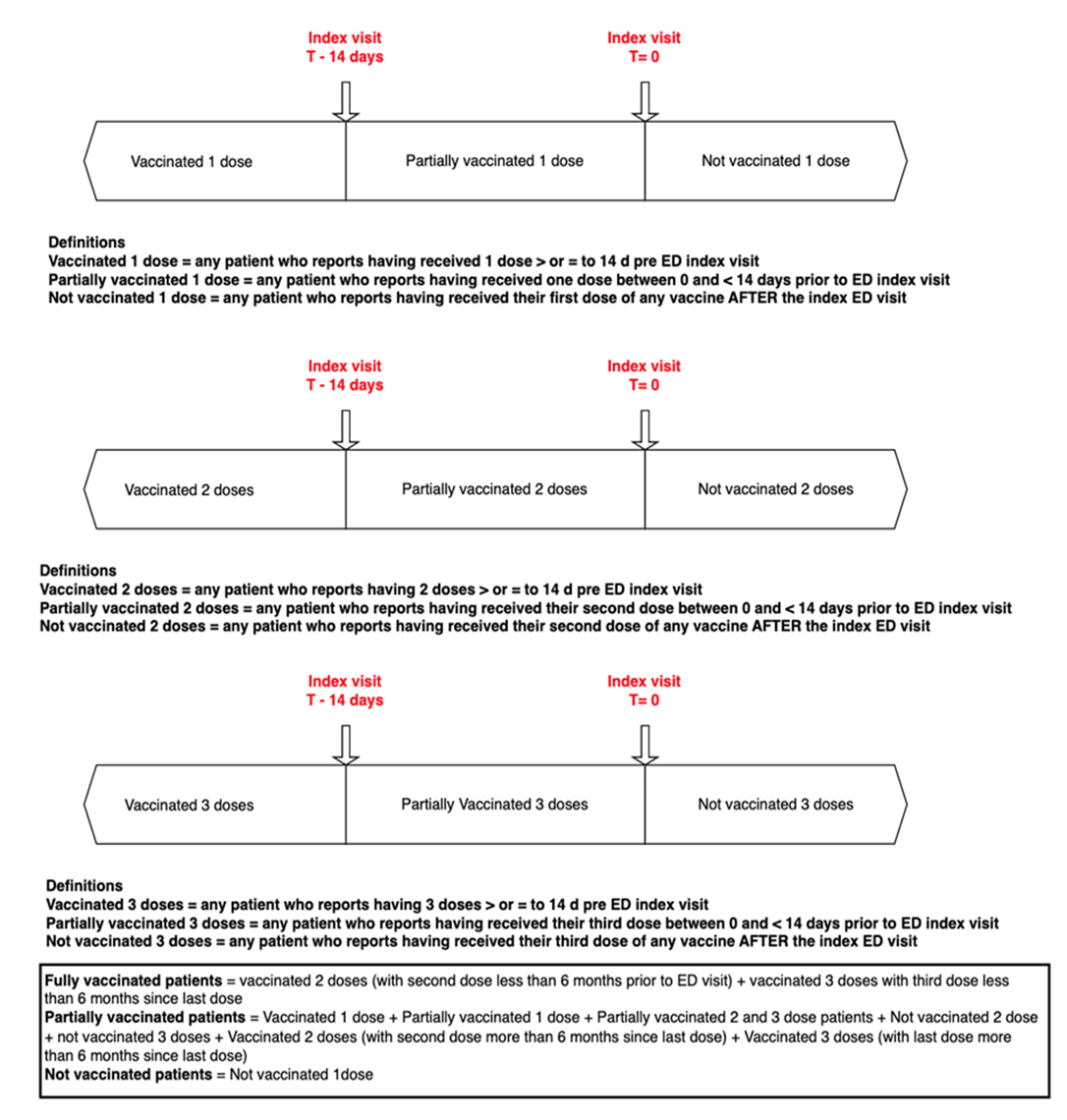

Supplement: Multimedia Appendix 1 [file publichealth_v9i1e44465_app1.png]
