# Supplementary material for: Accuracy of Self-Reported COVID-19 Vaccination Status Compared With a Public Health Vaccination Registry in Québec: Observational Diagnostic Study
Source: JMIR Public Health Surveill. 2023 Jun 16;9:e44465. doi: 10.2196/44465 (PMC10278735; doi:10.2196/44465)
Supplement: Multimedia Appendix 2 [file publichealth_v9i1e44465_app2.docx]

Multimedia Appendix 2. Sociodemographic and clinical characteristics of all study participants by concordant or discordant vaccination status.

|  | | **Eligible participants who consented for phone follow-up with final vaccination status available**  **(N=1361)** | **Participants with concordant vaccination status**  **(n=1308)** | **Participants with discordant vaccination status**  **(n=53)** |
| --- | --- | --- | --- | --- |
|  | |  |  |  |
| **Age (years), mean (SD)** | | 55.1 (17.4) | 55.3 (17.3) | 51.1 (20.5) |
| **Age group (years), n (%)** | |  |  |  |
|  | < 24 years | 45 (3.3) | 40 (3.1) | 5 (9.4) |
|  | 25-34 years | 230 (16.9) | 216 (16.5) | 14 (26.4) |
|  | 35-64 years | 685 (50.3) | 668 (51.1) | 17 (32.1) |
|  | 65-79 years | 272 (20.0) | 259 (19.8) | 13 (24.5) |
|  | 80+ years | 129 (9.5) | 125 (9.5) | < 5 |
| **Sex, n (%)** | |  |  |  |
|  | Male | 686 (50.4) | 659 (50.4) | 27 (50.9) |
|  | Female | 675 (49.6) | 649 (49.6) | 26 (49.1) |
| **Self-reported gender, n (%)** | | 0 (0) |  |  |
|  | Man | 678 (49.8) | 652 (49.8) | 26 (49.1) |
|  | Woman | 673 (49.4) | 646 (49.4) | 27 (50.9) |
|  | Two-spirit | < 5 | < 5 | 0 (0) |
|  | Prefer not to answer | 9 (0.7) | 9 (0.7) | 0 (0) |
| **Site, n (%)** | |  |  |  |
|  | Hôtel-Dieu de Lévis | 293 (21.5) | 284 (21.7) | 9 (17.0) |
|  | Royal Victoria | 501 (36.8) | 482 (36.9) | 19 (35.8) |
|  | Montreal General | 132 (9.7) | 126 (9.6) | 6 (11.3) |
|  | Sacré-Coeur de Montréal | 435 (32.0) | 416 (31.8) | 19 (35.8) |
| **Vaccination status**  **in vaccine registry, n (%)** | |  |  |  |
|  | Yes (at least one dose) | 913 (67.1) | 896 (68.5) | 17 (32.1) |
|  | No (no doses given) | 448 (32.9) | 412 (31.5) | 36 (67.9) |
| **Vaccination status**  **Self-reported, n (%)** | |  |  |  |
|  | Yes (at least one dose) | 932 (68.5) | 896 (68.5) | 36 (67.9) |
|  | No (no doses given) | 429 (31.5) | 412 (31.5) | 17 (32.1) |
| **Vaccine brand**  **in vaccine registry, n (%)** | |  |  |  |
|  | Pfizer-BioNTech | 707 (51.9) | 690 (52.8) | 17 (32.1) |
|  | Moderna | 162 (11.9) | 162 (12.4) | 0 (0) |
|  | AstraZeneca/Covishield | 42 (3.1) | 42 (3.2) | 0 (0) |
|  | Johnson & Johnson | < 5 | < 5 | 0 (0) |
|  | Not vaccinated | 448 (32.9) | 412 (31.5) | 36 (67.9) |
| **Vaccine brand**  **Self-reported, n (%)** | |  |  |  |
|  | Pfizer-BioNTech | 689 (50.6) | 666 (50.9) | 23 (43.4) |
|  | Moderna | 158 (11.6) | 148 (11.3) | 10 (18.9) |
|  | AstraZeneca/Covishield | 42 (3.1) | 40 (3.1) | < 5 |
|  | Johnson & Johnson | < 5 | < 5 | 0 (0) |
|  | Unknown, but vaccinated | 37 (2.7) | 37 (2.8) | < 5 |
|  | Not vaccinated | 433 (31.8) | 415 (31.7) | 17 (32.1) |
| **Number of vaccines dose in vaccine registry, n (%)** | |  |  |  |
|  | 0 | 448 (32.9) | 412 (31.5) | 36 (67.9) |
|  | 1 | 366 (26.9) | 359 (27.4) | 7 (13.2) |
|  | 2 | 501 (36.8) | 493 (37.7) | 8 (15.1) |
|  | 3 | 46 (3.4) | 44 (3.4) | < 5 |
| **Number of vaccines –**  **Self-reported, n (%)** | |  |  |  |
|  | 0 | 429 (31.5) | 412 (31.5) | 17 (32.1) |
|  | 1 | 368 (27.0) | 348 (26.6) | 20 (37.7) |
|  | 2 | 511 (37.5) | 498 (38.1) | 13 (24.5) |
|  | 3 | 53 (3.9) | 50 (3.8) | < 5 |
| **Household size, n (%)** | |  |  |  |
|  | 1-2 | 698 (51.3) | 668 (51.1) | 30 (56.6) |
|  | 3-4 | 457 (33.6) | 441 (33.7) | 16 (30.2) |
|  | 5-7 | 177 (13.0) | 172 (13.1) | 5 (9.4) |
|  | 8 or more | 15 (1.1) | 14 (1.1) | < 5 |
|  | Prefer not to answer | 14 (1.0) | 13 (1.0) | < 5 |
| **Children, n (%)** | |  |  |  |
|  | Yes | 442 (32.5) | 426 (32.6) | 16 (30.2) |
|  | No | 908 (66.7) | 872 (66.7) | 36 (67.9) |
|  | Prefer not to answer | 11 (0.8) | 10 (0.8) | < 5 |
| **Caregiver/Assistance, n (%)** | |  |  |  |
|  | Yes | 131 (9.6) | 125 (9.6) | 6 (11.3) |
|  | No | 1220 (89.6) | 1174 (89.8) | 46 (86.8) |
|  | Prefer not to answer | 10 (0.7) | 9 (0.7) | < 5 |
| **Quarantine, n (%)** | |  |  |  |
|  | Yes | 1201 (88.2) | 1155 (88.3) | 46 (86.8) |
|  | No | 80 (5.9) | 76 (5.8) | < 5 |
|  | Prefer not to answer | 80 (5.9) | 77 (5.9) | < 5 |
| **Race, n (%)** | |  |  |  |
|  | Arab/Middle Eastern | 191 (14.0) | 184 (14.1) | 7 (13.2) |
|  | Black | 164 (12.0) | 154 (11.8) | 10 (18.9) |
|  | East Asian/  Southeast Asian | 42 (3.1) | 42 (3.2) | 0 (0) |
|  | Indigenous | 8 (0.6) | 8 (0.6) | 0 (0) |
|  | Latin American | 59 (4.3) | 57 (4.4) | < 5 |
|  | South Asian | 80 (5.9) | 76 (5.8) | < 5 |
|  | White | 730 (53.6) | 704 (53.8) | 26 (49.1) |
|  | Other | 60 (4.4) | 56 (4.3) | < 5 |
|  | Prefer not to answer | 27 (2.0) | 27 (2.1) | 0 (0) |
| **Landed immigrant, n (%)** | |  |  |  |
|  | Yes | 656 (48.2) | 628 (48.0) | 28 (52.8) |
|  | No | 674 (49.5) | 650 (49.7) | 24 (45.3) |
|  | Prefer not to answer | 31 (2.3) | 30 (2.3) | < 5 |
| **Education level, n (%)** | |  |  |  |
|  | No certificate, diploma, or degree | 106 (7.8) | 102 (7.8) | < 5 |
|  | High school diploma or equivalency certificate | 323 (23.7) | 310 (23.7) | 13 (24.5) |
|  | Trade certificate or diploma College, CEGEP/College, or non-university certificate or diploma | 367 (27.0) | 356 (27.2) | 11 (20.8) |
|  | University certificate or diploma below bachelor level | 51 (3.7) | 51 (3.9) | 20 (37.7) |
|  | University certificate, diploma, or degree at bachelor level or above | 464 (34.1) | 444 (33.9) | 5 (9.4) |
|  | Prefer not to answer | 50 (3.7) | 45 (3.4) | < 5 |
| **Employment, n (%)** | |  |  |  |
|  | Employed | 796 (58.5) | 771 (58.9) | 25 (47.2) |
|  | Retired | 353 (25.9) | 338 (25.8) | 15 (28.3) |
|  | Unemployed | 191 (14.0) | 180 (13.8) | 11 (20.8) |
|  | Prefer not to answer | 21 (1.5) | 19 (1.5) | < 5 |
| **Income pre-COVID-19 pandemic, n (%)**^a^ | |  |  |  |
|  | <$22,440-29,900 | 186 (13.7) | 180 (13.8) | 6 (11.3) |
|  | $29,901-42,300 | 123 (9.0) | 121 (9.3) | < 5 |
|  | $42,301-55,300 | 91 (6.7) | 87 (6.7) | < 5 |
|  | $55,301-73,700 | 103 (7.6) | 100 (7.6) | < 5 |
|  | $73,701+ | 323 (23.7) | 317 (24.2) | 6 (11.3) |
|  | Prefer not to answer | 535 (39.3) | 503 (38.5) | 32 (60.4) |
| **Current income, n (%)^a^** | |  |  |  |
|  | <$22,440-29,900 | 211 (15.5) | 205 (15.7) | 6 (11.3) |
|  | $29,901-42,300 | 108 (7.9) | 106 (8.1) | < 5 |
|  | $42,301-55,300 | 80 (5.9) | 78 (6.0) | < 5 |
|  | $55,301-73,700 | 111 (8.2) | 107 (8.2) | < 5 |
|  | $73,701+ | 299 (22.0) | 294 (22.5) | 5 (9.4) |
|  | Prefer not to answer | 552 (40.6) | 518 (39.6) | 34 (64.2) |
| **Comorbid conditions, n (%)** | |  |  |  |
|  | Coronary Artery Disease | 99 (7.3) | 95 (7.3) | < 5 |
|  | Hypertension | 440 (32.3) | 424 (32.4) | 16 (30.2) |
|  | Asthma | 144 (10.6) | 140 (10.7) | < 5 |
|  | Diabetes | 241 (17.7) | 231 (17.7) | 10 (18.9) |
|  | Chronic neurological conditions | 66 (4.8) | 62 (4.7) | < 5 |
|  | Rheumatologic disorder | 66 (4.8) | 60 (4.6) | 6 (11.3) |
|  | Past malignancy | 73 (5.4) | 71 (5.4) | < 5 |
|  | Psychiatric Condition/  Mental Health Diagnosis | 113 (8.3) | 106 (8.1) | 7 (13.2) |
|  | Dyslipidemia | 314 (23.1) | 301 (23.0) | 13 (24.5) |
|  | Hypothyroidism | 160 (11.8) | 153 (11.7) | 7 (13.2) |
| **Illicit substance use, n (%)** | |  |  |  |
|  | Current/Past user | 15 (1.1) | 14 (1.1) | < 5 |
|  | Never user | 448 (32.9) | 429 (32.8) | 19 (35.8) |
|  | Unknown | 898 (66.0) | 865 (66.1) | 33 (62.3) |
| **Intubated in-hospital, n (%)** | |  |  |  |
|  | Yes | 10 (0.7) | 9 (0.7) | < 5 |
|  | No | 1351 (99.3) | 1299 (99.3) | 52 (98.1) |
| **ED disposition, n (%)** | |  |  |  |
|  | Discharged home | 740 (54.4) | 710 (54.3) | 30 (56.6) |
|  | Admitted | 576 (42.3) | 554 (42.4) | 22 (41.5) |
|  | Transferred to other hospital | 38 (2.8) | 37 (2.8) | < 5 |
|  | Transfer to LTC / Rehab | < 5 | < 5 | 0 (0) |
|  | Other | 6 (0.4) | 6 (0.5) | 0 (0) |
| **Days between first vaccine dose and consent, median [IQR]** | | 126 [62-196] | 126 [62-196] | 94 [53-191] |

^a^ The income data is presented as CAD $ (CAD $1.3 (US $1)).

SD: standard deviation; IQR: Interquartile range
